# Takayasu arteritis with congestive heart failure in 26-year-old male: a case report

**DOI:** 10.1186/s43044-022-00279-5

**Published:** 2022-05-21

**Authors:** Suryono Suryono, Pipiet Wulandari, Dwi Ariyanti, Aditha Satria Maulana, R. Handi Sembodo, Narendra Wahyu Junior, Antonius Dwi Saputra

**Affiliations:** 1 Departement of Cardiology, Faculty of Medicine, University of Jember - Dr. Soebandi General Hospital, Jl. Kalimantan No. 37, Jember, East Java 68121 Indonesia; 2 Faculty of Medicine, University of Jember, Jl. Kalimantan No. 37, Jember, East Java 68121 Indonesia; 3 Departement of Radiology, Faculty of Medicine, University of Jember - Dr. Soebandi General Hospital, Jl. Kalimantan No. 37, Jember, East Java 68121 Indonesia

**Keywords:** Takayasu arteritis, Congestive heart failure, Echocardiography, Computed tomography angiography, Case report

## Abstract

**Background:**

Takayasu arteritis (TA) is included in large vessel vasculitis with unknown aetiopathogenesis. TA is one of the rare diseases with a predilection for young women. The diagnosis of TA is difficult due to variation in clinical presentations and non-specific initial symptoms. This case demonstrates rare TA in a young male with congestive heart failure as the predominant manifestation.

**Case presentation:**

We report a 26-year-old male presented with severe dyspnea, palpitation, orthopnea, paroxysmal nocturnal dyspnea, and claudication in the left arm. Four limbs blood pressure discrepancy was present. Chest X-ray showed cardiomegaly with calcification aortic arch and pulmonary edema. Echocardiography revealed that left ventricular ejection fraction decreased with severe aortic and mitral valve regurgitation. Computed tomography angiography showed stenosis of the left common carotid artery and total occlusion of the left subclavian artery with collateral artery. There was vascular thickness and calcification from the peri-aortic valve, ascending aorta, aortic arch, and thoracic descending aorta until abdominal aorta with high-grade stenosis on the inferior side of the renal artery branching accompanied by a post-stenotic dilatation.

**Conclusions:**

This patient’s heart failure was precipitated by secondary hypertension and aortic regurgitation caused by vasculitis of TA. In general, there is no difference in the management of congestive heart failure in patients with TA. Optimized pharmacology therapy with combination steroid and methotrexate successfully inducing remission of TA after 3-months follow-up.

## Background

Takayasu arteritis (TA) is characterized by chronic granulomatous inflammation of the blood vessel walls and is included in large vessel vasculitis with an unknown aetiopathogenesis. TA is also called ‘occlusive thromboarthropathy’, ‘Martorell syndrome’, or ‘pulseless disease’. TA mainly involves large vessels such as ascending aorta, aortic arch, and its main branches, descending aorta, abdominal aorta, and renal arteries. TA is a rare disease that mainly affects young females and commonly in the second and third decades of life [1, 2].

The diagnosis of TA is difficult due to variation in clinical presentations and non-specific initial symptoms. Hypertension, limb claudication, light-headedness, fever, weight loss, arthralgia, and arterial pain are common manifestations [2]. Several cases have been reported with various clinical manifestations. This case demonstrates rare TA in a young male with congestive heart failure as the predominant manifestation.

## Case presentation

A 26-year-old male presented with severe dyspnea, palpitation, orthopnea, and paroxysmal nocturnal dyspnea. He had hypertension, medical history of ischemic stroke 5 months ago, and repeated hospitalization caused by congestive heart failure. His blood pressure in the right arm was 180/64 mmHg. The heart rate was irregular with 150 beats/min. The jugular venous pulse was elevated to 9 cm of water. There was murmur systolic on intercostal space (ICS) 5 anterior axilla line (AAL) sinistra and murmur diastolic on ICS 2 parasternal line (PSL) dextra. Respiratory rate of 28 breaths/min, oxygen saturation at the right arm was 92% without supplemental oxygen, and body mass index of 19,5 kg/m^2^.

**Fig. 1:**
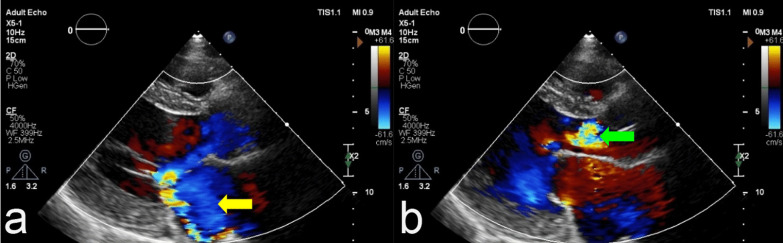
Echocardiogram showed left atrial and ventricular dilatation with severe **a** mitral valve regurgitation (yellow arrow) and **b** aortic valve regurgitation (green arrow)

An electrocardiogram showed atrial fibrillation (AF) with a rapid ventricular response. A routine laboratory examination did not show any abnormality. Chest X-ray showed cardiomegaly with calcification aortic arch and pulmonary edema. Echocardiography found that left ventricular ejection fraction (LVEF) was decreased (48,5%) with left atrial and ventricular dilatation. Echocardiography also found severe aortic and mitral valve regurgitation (Fig. 1). He was diagnosed with congestive heart failure with mildly reduced ejection fraction (HFmrEF) and classified in New York Heart Association (NYHA) class IV.

**Fig. 2:**
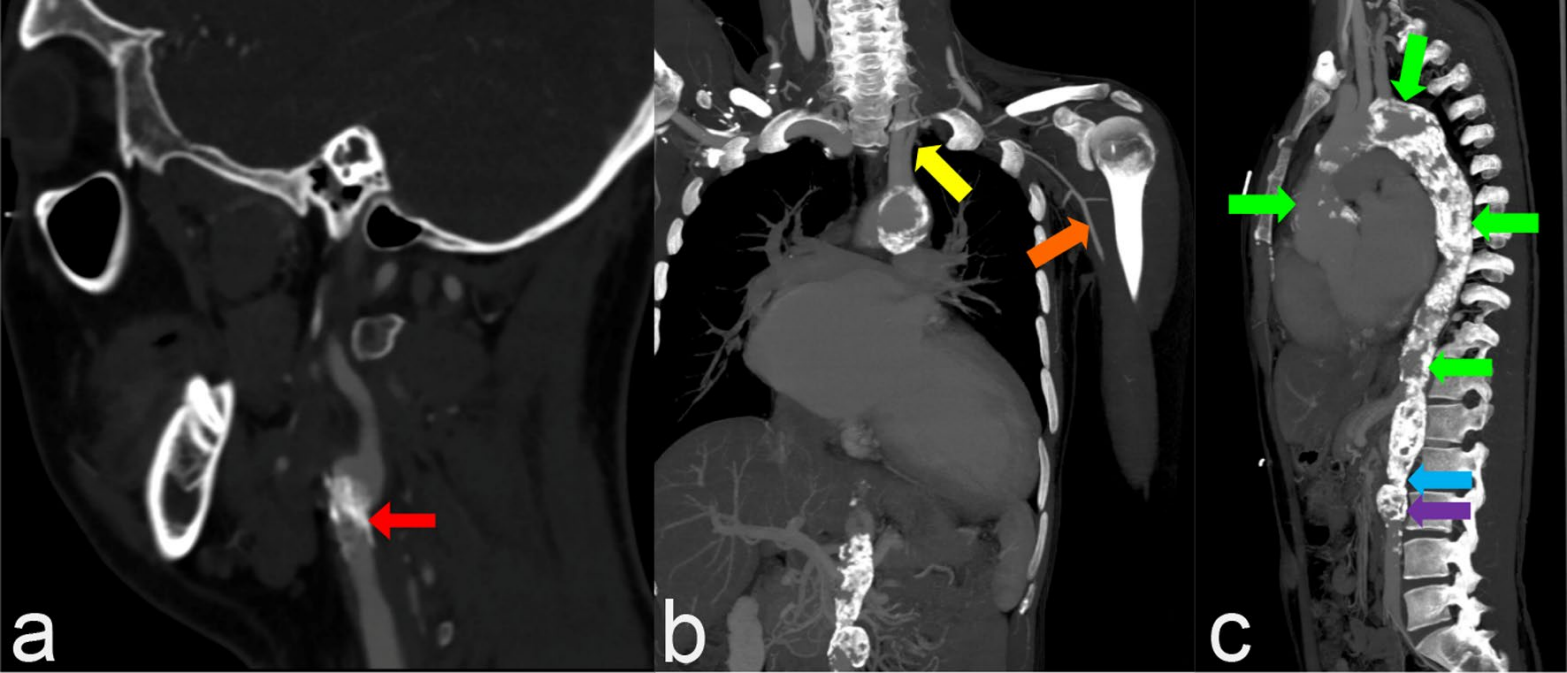
CTA examination showed **a** calcification in the left common carotid artery (red arrow) leading to stenosis; **b** total occlusion of the left subclavian artery (yellow arrow) with the left axillary artery (orange arrow) receiving vascularization from the collateral artery of the common carotid artery; (**c**) calcification of the peri-aortic valve, ascending aorta, aortic arch, descending aorta, and abdominal aorta (green arrow) with high-grade stenosis on the inferior side of the renal artery branching (blue arrow) accompanied by a post-stenotic dilatation (purple arrow)

After one day of hospitalization, the patient also complained of intermittent claudication in the left arm. Clinical examination found an absence of the left brachial pulse and the left carotid artery bruit. Four limbs blood pressure discrepancy was present (right arm 163/50 mmHg, left arm 96/55 mmHg, right leg 90/60 mmHg, left leg 95/55 mmHg). Computed tomography angiography (CTA) showed calcification in the left common carotid artery leading to stenosis and total occlusion of the left subclavian artery with the collateral artery branch from the left common carotid artery that supplies vascularization of the left arm. There was vascular thickness and calcification from the peri-aortic valve, ascending aorta, aortic arch, and thoracic descending aorta until abdominal aorta with high-grade stenosis on the inferior side of the renal artery branching accompanied by a post-stenotic dilatation (Fig. 2). Inflammatory markers evaluation that needed notable are elevation C-reactive protein (CRP) level (45,1 mg/L) and erythrocyte sedimentation rate (ESR) level (25 mm/h). Other laboratory evaluations were antinuclear antibody (ANA) titer, antistreptolysin O (ASO) titer, and interferon-gamma release assay (IGRA) showed negative results. He has subsequently diagnosed with Takayasu arteritis according to the American College of Rheumatology criteria.

For treatment of hypertension and HFmrEF, beta-blocker bisoprolol 2,5 mg/day, angiotensin-converting enzyme inhibitor (ACE-I) ramipril 5 mg/day, and mineralocorticoid receptor antagonists (MRA) spironolactone 25 mg/day therapy were initiated. Loop diuretic furosemide 40 mg/day is given to treat congestive symptoms of heart failure and glycoside digoxin 0,25 mg/day is given as rate control of atrial fibrillation. Anticoagulant warfarin 2 mg/day was given to prevent thromboembolism. A combination of methotrexate 7,5 mg/week and high-dose methylprednisolone at 48 mg/day with subsequent tapering was given for induction of remission to inhibition vasculitis of TA. After 3 months of high-dose steroid and methotrexate therapy, the patient showed a decrease in symptoms of NYHA class II for heart failure and a decrease in the frequency of rehospitalization. The patient also did not complain of claudication in the left arm. On physical examination, the patient's right arm blood pressure was 146/55 mmHg and the left arm was 105/50 mmHg. Follow-up evaluation of inflammatory markers CRP and ESR showed normal results.

## Discussion

The prevalence of Takayasu arteritis is very rare with an incidence in the western population only 1.2–2.6 per million per year. However, the highest prevalence of TA has been observed in Japan reaching 40 per million. TA generally 80–90% affects females [2, 3]. The incidence of TA in Indonesia is currently unknown, but several cases have been reported with various clinical manifestations. Lusida et al. reported a case of TA in an 18-year-old woman with recurrent fever and chronic malaise accompanied by headache and claudication of the hands and feet and postprandial abdominal discomfort [4]. The most recent case of TA was reported by Akbar et al., in a 19-year-old woman with ischemic stroke, right hemiplegia, extremity claudication, and decreased left brachial artery pulse [5]. We report a case of rare TA in a young male with clinical manifestation predominantly congestive heart failure and claudication of the left hand.

Takayasu arteritis etiology is believed to be an autoimmune process and correlated with the human leukocyte antigens (HLA) such as A2, A9, B35, B52, and DR4. In Japanese and other populations has been established that HLA-B52 had the strongest association. A worse prognosis appears to be conveyed by HLA-B52 positive Japanese patients [6]. A rise in the pro-inflammatory cytokine concentrations in serum plays an important role in the pathogenesis such as interleukin (IL)-6, IL-8, IL-9, IL-12, IL-17A, IL-18, interferon (IFN)-γ, and tumor necrosis factor (TNF)-α [6, 7]. There is an association between TA and Mycobacterium tuberculosis, previous study found 110 tuberculosis cases out of 669 cases of TA. TA patients who are candidates for immunosuppressive therapy are advised to screen for latent TB [8]. Molecular mimicry between the human 65-kDa heat shock protein (HSP) and mycobacterial 65-kDa HSP has been suggested to lead to an autoimmune response [6]. In our case, the etiology of TA in the patient is still not known. There is no relationship between TA with other autoimmune diseases shown by the negative result of the ANA test. IGRA test also showed negative results. This result revealed patient has not been exposed to tuberculosis bacterial infection.

The clinical manifestations of Takayasu arteritis vary from asymptomatic, non-specific symptoms to severe symptoms. There are three stages in TA, first is the ‘early systemic or pre-pulseless stage’ with non-specific signs and symptoms of inflammation such as mild fever, night sweats, malaise, joint pains, muscle ache, fatigue, and loss of weight. During this stage, the disease course may follow remitting/relapsing which makes diagnosis difficult. After several months or years, the second stage is ‘vascular inflammatory’ characterized by symptoms of vascular inflammation and vascular insufficiency will become apparent, such as pain over vessels (angiodynia), hypertension, arterial bruits, limb claudication, ocular, dermatologic, and neurologic manifestations. The last stage is ‘settles down or burns out stage’, this stage refers to fibrosis occurrence. This stage does not occur in all patients and it is associated with remission [2, 3, 6]. Heart failure in TA can be caused by secondary hypertension, systemic arterial involvement, pulmonary vascular involvement, and acute or chronic AR in patients with TA. A previous study found cardiac valve involvement in 34.9% of the TA patients, with AR being the common lesion in TA patients. AR develops primarily as a result of annular dilatation, inflammation rarely involves the aortic valve. Chronic AR leads to heart failure that causes death in patients with TA [9].

We report an atypical manifestation of Takayasu arteritis in a young male with dyspnea, orthopnea, and paroxysmal nocturnal dyspnea caused by pulmonary edema and heart failure. Heart failure and aortic valve regurgitation may be led by secondary hypertension caused by vasculitis aorta of TA especially high-grade stenosis in the aorta abdominal and total occlusion of the left subclavian artery. Stenosis of the left common carotid artery may lead history of ischemic stroke 5 months ago. Aortic regurgitation also can be caused by peri-aortic valve calcification. There was no thickness and stiffness in the heart valves which indicated inflammation due to rheumatic fever disease. ASO examination also showed negative results. Mitral regurgitation (MR) can be caused by left ventricular dilatation. Claudication in the left arm of the patient may be led by total occlusion of the left subclavian artery but compensated by a collateral artery branch from the left common carotid artery that supplies vascularization of the left arm.

The diagnosis of Takayasu arteritis was made according to the American College of Rheumatology criteria (1990) consisting of: (a) age at disease onset < 40 years, (b) claudication of extremities, (c) decreased brachial artery pulse, (d) subclavian arteries or aorta bruit (e) blood pressure difference > 10 mmHg, (f) abnormality of angiography. The diagnosis of TA is established when any 3 of the above 6 criteria are present [1]. In this case, all criteria are present for the patient that established a diagnosis of TA.

Currently, the major way of monitoring Takayasu arteritis is based on the evaluation of conventional inflammation biomarkers, including serum erythrocyte sedimentation rate (ESR), C-reactive protein (CRP) level, and the imaging tests, such as angiography. However, some cases have obvious inflammatory symptoms with no significant increase in either CRP or ESR values, such as relapsed TA patients [10, 11]. In our case, there is an elevation of ESR and CRP levels that revealed inflammation and damage to blood vessels or organs. Inflammation of vessels may be revealed by magnetic resonance imaging (MRI) or Doppler ultrasound. Magnetic resonance angiography and CTA are used for the gold standard diagnosis of TA and evaluation of vascular lesions. In particular, pan-angiography allows a correct assessment of the extension of the disease which correlates with the severity of TA [1, 12]. In this case, we used CTA to establish the diagnosis of TA. According to the classification of TA from the Takayasu Conference 1994 by anatomic distribution (Table 1), the TA of the patient can be classified as type V revealing a combination of type IIb and type IV or vasculitis involving ascending aorta, aortic arch, and its branches, thoracic descending aorta with the abdominal aorta and/or renal arteries [1]. A previous large Japanese study of 1,372 patients showed that the most common angiographic types were I and V, type I was common in female patients, and type V in male patients [6].

**Table 1:** Classification of TA from the Takayasu Conference 1994 [1]

Type	Vessel involvement
Type I	Branches from the aortic arch
Type IIa	Ascending aorta, aortic arch and its branches
Type IIb	Ascending aorta, aortic arch and its branches, thoracic descending aorta
Type III	Thoracic descending aorta, abdominal aorta, and/or renal arteries
Type IV	Abdominal aorta and/or renal arteries
Type V	Combined features of types IIb and IV

Management of Takayasu arteritis involved medical and surgical treatment. Medical treatments purpose to induce remission, reduce active inflammation, and minimize arterial injury to prevent the development of vascular complications. EULAR (European League Against Rheumatism) guidelines recommend prednisolone as the first-line agent with an initial dose of 1 mg/kg/day (maximum 60 mg/day) and gradual tapering [1, 13]. The combination of steroid and methotrexate therapy is used as a steroid-sparing agent to control disease progression and minimize the risk of steroid-related complications. Especially when steroid treatment is stopped there is a considerable risk of relapse. Previous studies suggest azathioprine and methotrexate are effective to stop the progress of arterial lesions and inducing remission [1, 2]. For our patient, we used a combination of methotrexate 7,5 mg/week and high-dose methylprednisolone at 48 mg daily with gradual tapering.

According to the European Society of Cardiology (ESC) guidelines patients with an LVEF of 41% until 49% showed mildly reduced left ventricle systolic function can be diagnosed with HFmrEF. In general, there is no difference in the management of congestive heart failure in patients with TA. Beta-blocker, ACE-I, and MRA are recommended for HFrEF treatment. Beta-blockers proved capable to minimize morbidity and mortality in patients with HFrEF. Loop diuretics are suggested as well to reduce the congestion signs or symptoms in patients. In this case, the patient has been given ACE-I ramipril 5 mg/day and beta-blocker bisoprolol 2,5 mg/day, MRA spironolactone 25 mg/day, and diuretic furosemide 40 mg/day to treat congestive heart failure. Atrial Fibrillation with a rapid ventricular rate is recommended to use Digoxin. Our patient also was given glycoside digoxin 0,25 mg/day. The patient also was given anticoagulant warfarin 2 mg/day. Except contraindicated, a long-term anticoagulant oral is recommended in all patients with HF and paroxysmal, persistent, or permanent AF for stroke and thromboembolic events prevention [14]. TA remission is the absence of clinical signs and symptoms caused by active large vessel vasculitis and normalization of ESR and CRP [13]. After 3 months of steroid and methotrexate therapy, the patient showed a decrease in symptoms of heart failure and there is no claudication of the left arm. Follow-up CRP and ESR evaluation also showed normal results. This result revealed successful remission of Takayasu arteritis. The patient is satisfied with the treatment and feels better. For 3 months, the patient also showed a decreased frequency of hospitalization.

## Conclusions

This case demonstrates a rare case of Takayasu arteritis in a young male with congestive heart failure as the predominant manifestation. This patient’s heart failure was precipitated predominantly by secondary hypertension and aortic regurgitation caused by vasculitis of Takayasu arteritis. In general, there is no difference in the management of congestive heart failure in patients with Takayasu arteritis. Optimized pharmacology therapy with a combination of steroid and methotrexate successfully induced remission of Takayasu arteritis after a 3-months follow-up.

## Data Availability

Not applicable.

## Abbreviations

TA: Takayasu arteritis
ICS: Intercostal space
AAL: Anterior axilla line
PSL: Parasternal line
AF: Atrial fibrillation
LVEF: Left ventricular ejection fraction
HfmrEF: Heart failure mildly reduced ejection fraction
NYHA: New York Heart Association
CTA: Computed tomography angiography
AR: Aortic regurgitation
MR: Mitral regurgitation
CRP: C-reactive protein
ESR: Erythrocyte sedimentation rate
ANA: Antinuclear antibody
ASO: Antistreptolysin O
IGRA: Interferon-gamma release assay
ACE-I: Angiotensin-converting enzyme inhibitor
MRA: Mineralocorticoid receptor antagonists
HLA: Human leukocyte antigens
IL: Interleukin
IFN: Interferon
TNF: Tumor necrosis factor
HSP: Heat shock protein
MRI: Magnetic resonance imaging
ESC: European Society of Cardiology
